# Deficiency of maize starch-branching enzyme i results in altered starch fine structure, decreased digestibility and reduced coleoptile growth during germination

**DOI:** 10.1186/1471-2229-11-95

**Published:** 2011-05-21

**Authors:** Huan Xia, Marna Yandeau-Nelson, Donald B Thompson, Mark J Guiltinan

**Affiliations:** 1 MARS Petcare US, 315 Cool Springs Boulevard, Franklin, Tennessee 37067, USA; 2 Department of Biochemistry, Biophysics & Molecular Biology, Iowa State University, Ames, Iowa 50011-3260, USA; 3 Department of Food Science, The Pennsylvania State University, University Park, Pennsylvania 16802-2504, USA; 4 Department of Horticulture, The Pennsylvania State University, University Park, Pennsylvania 16802-5807, USA

## Abstract

**Background:**

Two distinct starch branching enzyme (SBE) isoforms predate the divergence of monocots and dicots and have been conserved in plants since then. This strongly suggests that both SBEI and SBEII provide unique selective advantages to plants. However, no phenotype for the SBEI mutation, *sbe1a*, had been previously observed. To explore this incongruity the objective of the present work was to characterize functional and molecular phenotypes of both *sbe1a *and wild-type (Wt) in the W64A maize inbred line.

**Results:**

Endosperm starch granules from the *sbe1a *mutant were more resistant to digestion by pancreatic α-amylase, and the *sbe1a *mutant starch had an altered branching pattern for amylopectin and amylose. When kernels were germinated, the *sbe1a *mutant was associated with shorter coleoptile length and higher residual starch content, suggesting that less efficient starch utilization may have impaired growth during germination.

**Conclusions:**

The present report documents for the first time a molecular phenotype due to the absence of SBEI, and suggests strongly that it is associated with altered physiological function of the starch *in vivo*. We believe that these results provide a plausible rationale for the conservation of SBEI in plants in both monocots and dicots, as greater seedling vigor would provide an important survival advantage when resources are limited.

## Background

The starch granule is a highly-ordered structure with alternating crystalline and amorphous growth rings [1,2]. Starch molecules are biopolymers of anhydroglucose units linked by α-1,4 and α-1,6 glycosidic bonds. They are composed of two glucan polymers, the generally linear fraction, amylose, and the branched fraction, amylopectin. The constituent amylopectin chains can be mainly categorized into A chains (not bearing any branches) and B chains (bearing one or more branches) [3]. The main physiological functions of starch include high-density storage of energy and the controlled release of this energy during starch degradation.

Starch-branching enzyme (SBE) plays an important role in starch biosynthesis by introducing branch points, the α-1,6 linkages in starch. Boyer and Preiss [4] identified three major SBE isoforms in developing maize kernels: SBEI, SBEIIa, and SBEIIb. The SBE isoforms have been shown to be encoded by different genes [5-8]. Phylogenetic analyses of SBE sequences from a number of plant species have shown that the SBEI and SBEII isoforms are conserved among most plants, and that SBEIIa and SBEIIb isoforms are conserved among most monocots [9-13]. Furthermore, genes belonging to both the SBEI and SBEII families can be identified in various lineages of green alga, which supports the theory that these two families of genes evolved approximately a billion years ago [14]. These examples of extreme evolutionary conservation are strong evidence for a specific and vital role for each enzyme isoform in starch biosynthesis.

*In vitro *biochemical analyses have documented that the SBEI and SBEII isoform activities are not identical [15,16], but these studies do not necessarily indicate their action *in vivo*, as starch biosynthesis occurs in the presence of starch synthases and debranching enzymes. Studies have suggested that multi-protein starch synthesizing complexes exist, and that interactions within these complexes could modulate the intricate structure of a developing starch granule [17-33]. Whether there are functional differences among SBE isoforms *in vivo *remains to be addressed.

Insight into a possible *in vivo *function of an SBE may be gained from the study of *sbe *mutants deficient in one or more SBE isoform activities. The maize *amylose extender *(*ae*) mutant, which is deficient in SBEIIb, has a profound effect on starch structure, leading to an increased amylose proportion and a reduced branching density of endosperm amylopectin [5,33-35]. More recently, studies of a maize *sbe2a *mutant showed that deficiency of the SBEIIa isoform decreased plant fitness and resulted in lower kernel yield, but there was minimal effect on kernel starch properties [11,36]. Previous work showed no effect of SBEI deficiency (in the *sbe1a *mutation) on starch molecular size and on chain length distribution after debranching [26,37]. Subsequently, preliminary analysis of susceptibility of *sbe1a *endosperm starch to pancreatic α-amylase digestion, using the AOAC procedure (2002.02) to determine enzyme-resistant starch (RS), indicated that *sbe1a *mutant endosperm starch had a greater resistance to digestion [38]. We reasoned that it was likely that the deficiency in SBEI led to reduced susceptibility to enzymatic digestion by altering the starch structure in some way. Thus, in this work we sought to confirm this initial observation and to explore more subtle aspects of starch structure in the *sbe1a *mutant. The objective of the present work was to characterize functional and molecular phenotypes of both *sbe1a *and wild-type (Wt) in the W64A maize inbred line.

## Results

### Starch Molecular Structure

**Figure 1:**
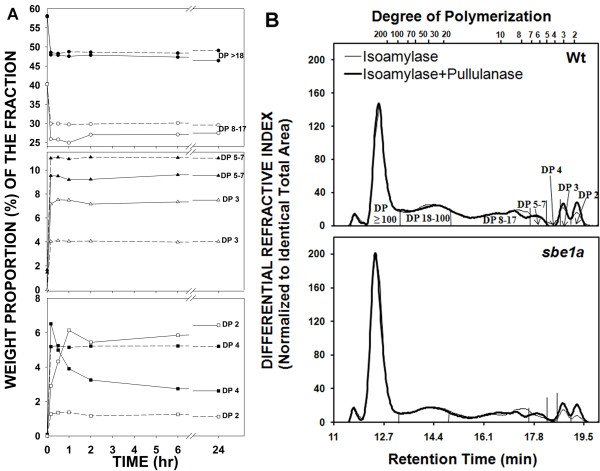
**Amylopectin and amylose structure of Wt and *sbe1a *mutant starch samples by HPSEC analysis**. **A**. Proportions of chains^1 ^from debranched^2 ^β-dextrins during time course of β-amylolysis of amylopectin from Wt (----) and *sbe1a *mutant (- - -) starch using β-amylase (250 U/mL). **B**. Chromatograms^1 ^of isoamylase-debranched and isoamylase-plus-pullulanase-debranched β-limit dextrins^3 ^from amylose fraction from Wt and *sbe1a *mutant starch. ^*1*^*Chromatographic regions were divided as in *[40]*. Proportions of DP ≥ 18, DP 8-17, DP 5-7, DP 4, DP 3 and DP 2 were calculated as the areas for DP ≥ 17.5, 7.5 ≤ DP ≤ 17.5, 4.5 ≤ DP ≤ 7.5, 3.5 ≤ DP ≤ 4.5, 2.5 ≤ DP ≤ 3.5, and DP ≤ 2.5, respectively, as in *[40]*. Proportions of chains in each region for **B **are presented in Additional File *4. *Calculation was based on representative chromatograms for starch from one biological replication. Values are percentage by weight*. ^*2*^*Debranching was performed successively with isoamylase for 24 h and pullulanase for 24 h*. ^*3*^*β-Limit dextrin was obtained after 3 times of 24-h β-amylolysis on amylose*.

To study the functional role of SBEI on molecular structure of amylose and amylopectin, Wt *Sbe1a *starch and mutant *sbe1a *starch were fractionated from mature kernels. The maize *sbe1a *mutant contains a *Mu *transposon in the 14th exon of the *Sbe1a *gene, and was previously shown to be null for the expression of SBEI transcript and protein [37]. The proportions, iodine binding properties, and size-exclusion chromatograms for the amylopectin and amylose fractions were similar for Wt and *sbe1a *starch (data not shown). To study the molecular fine structure, β-amylolysis and subsequent isoamylase and pullulanase debranching were applied to both the amylopectin and amylose fractions from Wt and *sbe1a*. Despite a similar chain length (CL) profile observed for both fractions from the two genotypes (see Additional File 1 online), the CL distribution after various extents of β-amylolysis showed differences for Wt and *sbe1a *(Figure 1A; see Additional File 2 online).

For the amylopectin fraction from both genotypes, hydrolysis with β-amylase caused a dramatic change in CL distribution within the first 10 min (Figure 1A): A major increase was observed below degree of polymerization (DP) ~10. In this region for Wt, the change in the CL distribution from 10 min to 24 h of β-amylolysis was primarily a reduction of the DP 4 stubs to DP 2 stubs; however, for the *sbe1a *sample no further reduction in DP 4 was observed after 10 min (Figure 1A). After 24 h of β-amylolysis, conditions necessary to produce β-limit dextrin (β-LD) [39,40], the *sbe1a *sample had a much smaller proportion of the DP 2 chains and a much larger proportion of DP 4 chains than the Wt sample (Figure 1A; see Additional File 2 &3 online).

For the amylose fraction from both genotypes, β-LD was produced. Analysis of the CL distribution of isoamylase-debranched β-LDs showed a higher proportion of chains of DP ≥ 100 and lower proportions of other chains (DP < 100), before and after pullulanase addition (Figure 1B; see Additional File 4 online). The subsequent pullulanase debranching led to an increase in both the DP 3 and DP 2 areas for both genotypes, and this increase was greater in *sbe1a *(Figure 1B; see Additional File 4 online). The subsequent pullulanase debranching also led to a decrease in chains of approximately DP 8-9 for both genotypes (Figure 1B).

### Starch Digestibility *In vitro *by Pancreatic α-Amylase

Starch hydrolysis is an important feature of starch function both in the plant and when the plant is used for human food. Hydrolysis of starch ingested as food can vary both with respect to the rate and the extent of digestion by pancreatic α-amylase. In the human digestive tract, the undigested starch that reaches the colon is termed RS; the level of RS is a measure of the extent of digestion by this enzyme. An official *in vitro *method (AOAC 2002.02) is used for determination of the RS level. This method was modified to allow study of both the digestion rate and the extent of digestion [41,42]. F-tests performed for a fully nested analysis of variance (ANOVA) showed an effect of genotype (*p *= 0.000), but no effect of biological replication (*p *= 0.334). The RS value was higher in the *sbe1a *mutant starch (13.2%) than in the Wt starch (1.6%) from measures of 3 biological replications (*p *< 0.05).

**Figure 2:**
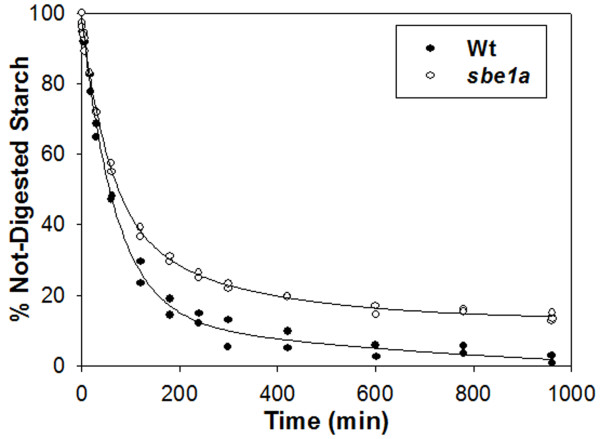
**Time-course of digestion of the resistant starch assay for Wt and *sbe1a *mutant starch**. Results shown were from one biological replication. Curves shown are best fits of analysis of combined data from two independent digestions.

The digestion pattern was similar among the three biological replications for each genotype (data not shown). For graphic illustration of the digestion time-course, curves for one biological replication for each genotype are shown in Figure 2. The kinetics of digestion were analyzed using a five-parameter, double-exponential decay model (see "Materials and Methods"), and the calculated parameters are presented in Table 1. A higher *y_0 _*(the limit of digestion as determined using the model) was found in *sbe1a *than for Wt (Table 1), consistent with the higher limit of digestion given by the RS value for this genotype.

**Table 1:** Kinetics of digestion^1 ^of the resistant starch assay for Wt and *sbe1a *mutant starch^2^

Starch	***y***_***0***_**(%)**	***S***_***1***_**(%)**	***k***_***1 ***_**(min**^**-1**^**)**	***S***_**2**_**(%)**	***k***_**2 **_**(min**^**-1**^**)**
**Wt**	-5.4 ± 2.3^a^	85.9 ± 3.5^b^	1.4 ± 0.1^a ^(×10^-2^)	17.9 ± 5.4^a^	0.9 ± 0.2^a ^(×10^-3^)
***sbe1a***	13.7 ± 2.8^b^	59.8 ± 3.0^a^	1.8 ± 0.1^b ^(×10^-2^)	24.3 ± 2.4^a^	3.0 ± 1.1^b ^(×10^-3^)

^1 ^Kinetic parameters are obtained from model fit using the double exponential decay equation:  where *y *is % NDS, *x *is the time, *y*_*0 *_is the y-value that the model asymptotically approaches, *S*_*1 *_and *S*_*2 *_are the concentrations of the two different substrate components, and *k*_*1 *_and *k*_*2 *_are the reaction rate constants for the decay of the two different components.

^2 ^Values are expressed as mean ± SD for three biological replications. Values for each biological replication were obtained from fit of combined data from two independent digestions. Significant differences (*p *< 0.05) in the same column, as determined by one-way ANOVA analysis, are indicated by different superscripts.

### Granular Morphology of Native Starch and Residual Starch after Digestion

**Figure 3:**
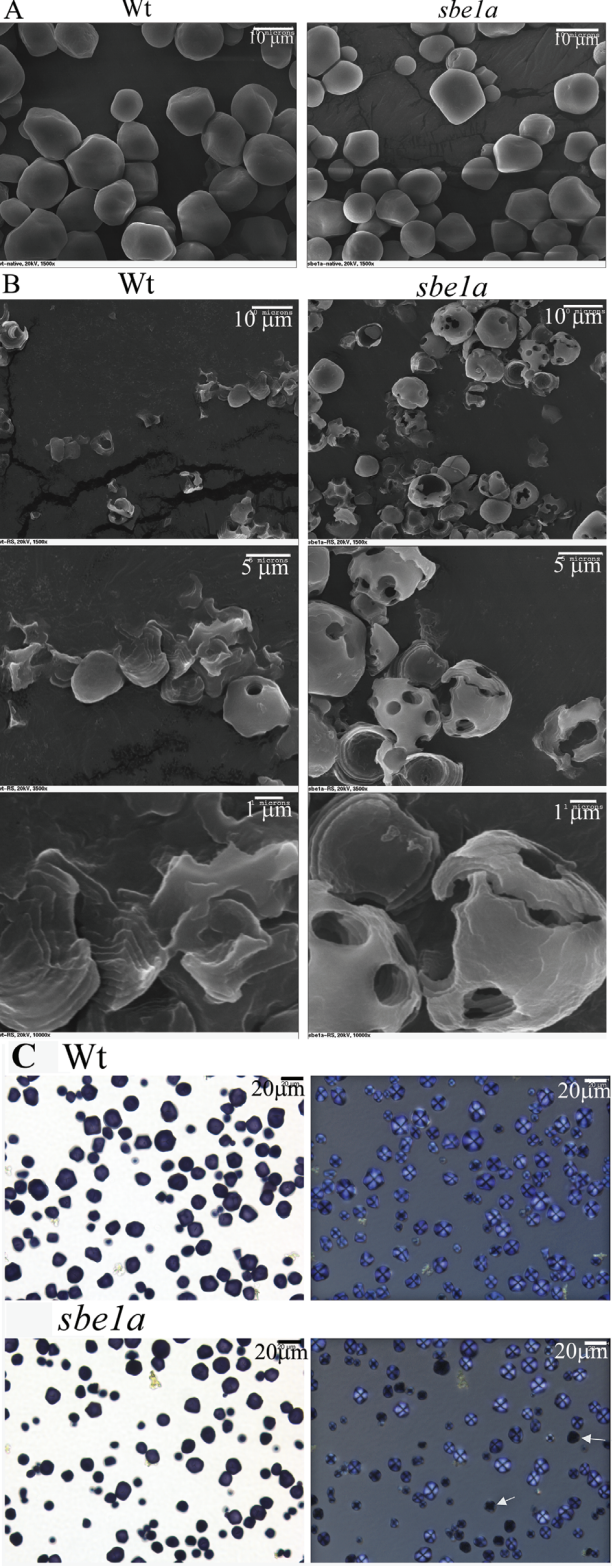
**Micrographs of Wt and *sbe1a *mutant starch samples**. **A**. Scanning electron micrographs of native starch from Wt (left) and *sbe1a *mutant (right). Scale bars represent 10 μm at the top of the graphs. **B**. Scanning electron micrographs of residual starch after 16-h α-amylase digestion from Wt and *sbe1a *mutant. Scale bars represent 10 μm, 5 μm, or 1 μm at the top of the graphs. **C**. Bright field (left) and polarized light (right) micrographs of native Wt and *sbe1a *mutant starch. The specimen were stained with 0.04% iodine and viewed within 5 min. Arrows point to dark stained granules.

Scanning electron microscopy was used to image starch granules from Wt and *sbe1a *mutant plants in native form and after the 16 h *in vitro *digestion with pancreatic α-amylase for determination of the RS value. Prior to digestion, native starch granules from Wt and *sbe1a *had similar morphology (Figure 3A) with an average diameter of 10.2 μm for Wt and 9.8 μm for *sbe1a*. However, after digestion, differences were observed between the two genotypes (Figure 3B; see Additional File 5 &6 online). Samples of the *sbe1a *RS contained many residual granules with distinct holes in the surface and hollow interiors, whereas for Wt only small fragments of residual granules were seen (Figure 3B). The Wt fragments also showed evident alternating layers on the edge of the pieces, which was less evidently present in *sbe1a *samples (Figure 3B; see Additional File 6 online).

Light micrographs of iodine-stained native granules are shown in Figure 3C. For both genotypes, all native starch granules were stained blue and produced a characteristic Maltese Cross when viewed in the polarized light microscope; however, *sbe1a *native starch showed more heterogeneity in staining as compared to Wt, as there were more relatively dark-stained granules in *sbe1a *than in Wt native starch (24.3% and 8.7%).

### Starch Utilization during Kernel Germination

As endosperm starch from the *sbe1a *mutant has a lower susceptibility to pancreatic α-amylase, we suspected that the *sbe1a *endosperm starch might be less readily utilized during kernel germination. To study the effect of *sbe1a *on kernel germination, starch utilization and coleoptile growth during germination of Wt and *sbe1a *mutant kernels were examined.

**Figure 4:**
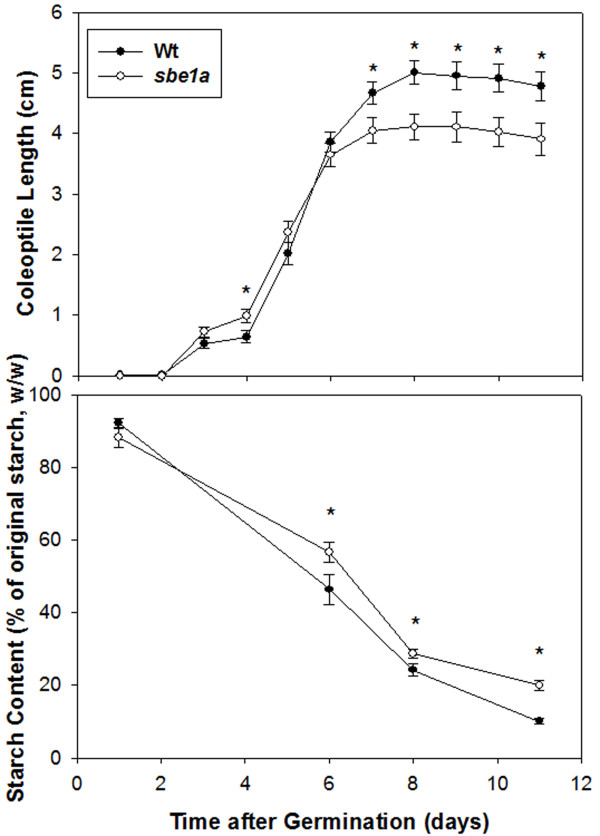
**Germination analysis of Wt and *sbe1a *mutant kernels**. The lengths of the emerged coleoptiles were measured on successive days during the incubation period^1^. Starch content in the germinating endosperm was quantified at Day 1, 6, 8, 11, and percentage of starch content at each day against the dry weight of Day 1 kernels was plotted^1^. ^1^Each data point is mean ± standard error of measurements of kernels from three biological replications. As 2 kernels were removed at Day 1, 6, 8, 11 for quantifying starch content, 15, 13, 11, and 9 kernels from three biological replications were used for coleoptile measurement Day 1, 2-6, 7-8, 9-11, respectively. Comparison between two genotypes for each day was made by one-way ANOVA analysis and a significant difference was marked by an asterisk (p < 0.05).

All the kernels from three different ears of both Wt and *sbe1a *genotypes were germinated, demonstrating no differences in germination rate. The coleoptile length of each genotype was measured daily over 11 days (Figure 4). The average length of *sbe1a *coleoptiles was shorter than Wt from Day 7 onward (Figure 4). For both genotypes the endosperm starch content decreased over time (Figure 4). On Days 6, 8, and 11, the starch content was higher in *sbe1a *germinating endosperm as compared to Wt, suggesting less utilization of starch. This trend is consistent with the reduced growth of *sbe1a *coleoptiles after Day 6.

## Discussion

### Starch Molecular Structure

In the present study, rapid degradation of chains DP ≥18 and DP 8-17 were observed for both Wt and *sbe1a *samples in the first 10 min of β-amylolysis (Figure 1A). As β-amylase cannot bypass branch points to hydrolyze starch chains, a plausible interpretation for the less extensive degradation of DP 8-17 in *sbe1a *would be that the B chains (those chains with other chains attached) [43] would have slightly longer internal segments and shorter external chains. For the second stage of β-amylolysis [44], a slow reduction in the amount of DP 4 chains was observed in Wt samples over the period of 10 min to 24 h but not in *sbe1a *samples (Figure 1A), suggesting differences in the proportion of branch points that would differentially limit access of the enzyme to glycosidic linkages [40].

**Figure 5:**
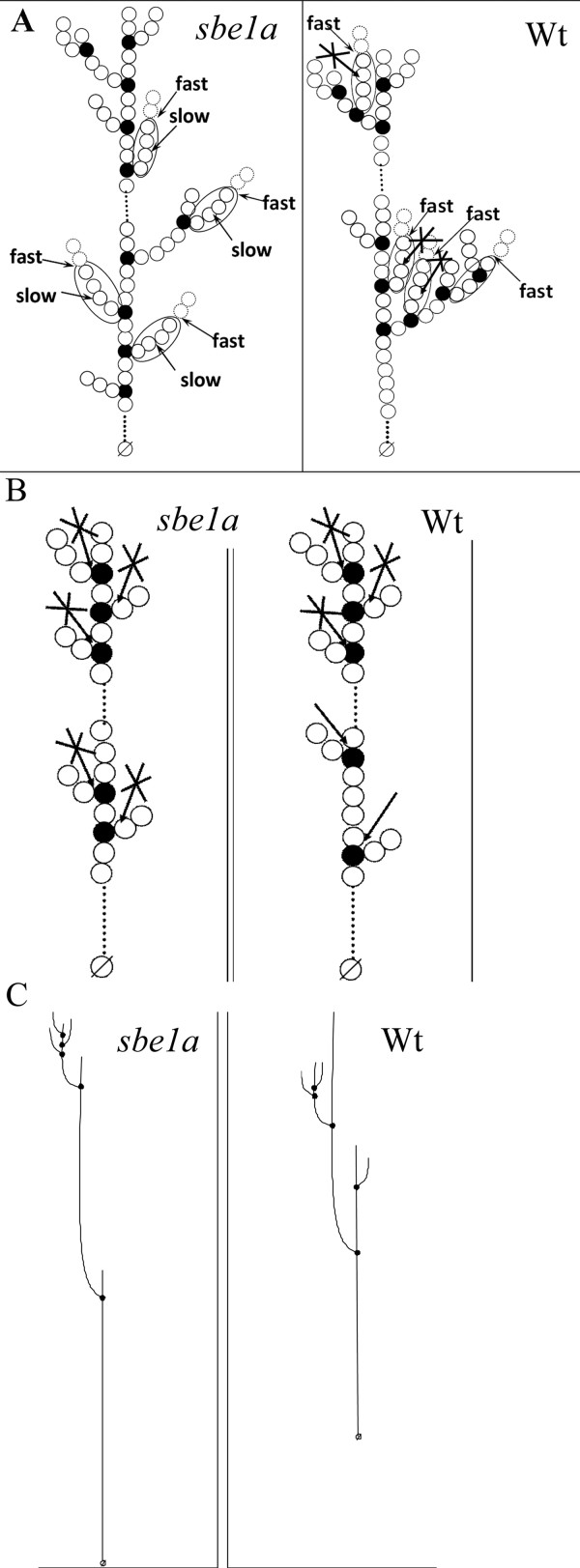
**Branching pattern models**. **A**. Branching pattern models for amylopectin from sbe1a and Wt starches. Shown are b-dextrins approaching the limit of digestion by β-amylase, with differences in the amount of DP 4 stubs. All circles indicate glucose units. Dotted line indicates more glucose units. Dotted circles indicate glucose hydrolyzed by β-amylase. Solid black circles indicate branch points. Circles with a slash indicate reducing ends. Circles in an ellipse indicate glucose units that would result in a DP 4 chain. Arrows indicate the action sites of β-amylase. Arrows with a cross indicates that action of β-amylase is prevented by closely associated branch points nearby. Fast and slow indicate the first and second stage of β-amylolysis, respectively. **B**. Branching pattern models for a region of the amylose from sbe1a and Wt starches. Shown are β-limit dextrins that are consistent with difference in action of isoamylase. All circles indicate glucose units. Dotted lines indicate more glucose units. Solid black circles indicate branch points. Circles with a slash indicate reducing ends. Arrows indicate the action sites of isoamylase. Arrows with a cross indicates that action of isoamylase is prevented by closely associated branch points nearby. The model does not consider the presence of B chains. **C**. Proposed overall amylose branching pattern models for sbe1a and Wt starches, consistent with the differences in actions of β-amylase and isoamylase. All lines indicate glucose chains. Solid black circles indicate branch points. Circles with a slash indicate reducing ends.

Amylopectin branching pattern models for both *sbe1a *and Wt are presented to account for this difference in β-amylase action on DP 4 stubs (Figure 5A). In the model for *sbe1a*, DP 4 stubs would be difficult for β-amylase to hydrolyze to DP 2 when closely associated branch points present a steric barrier to binding of β-amylase. Although most of the DP 4 is from residual A chains [43], some DP 4 chains from residual B chains would result from short B chains with short internal segments. The incomplete hydrolysis of DP 4 in *sbe1a *suggests that A chains are preferentially localized near another branch point, leading to 1) hindered hydrolysis of residual A chains of DP 4 to DP 2 due to steric constraint, and 2) more residual B chains with DP 4 due to incidence of short internal segments (Figure 5A). In the model for Wt, the DP 4 stubs would be slowly hydrolyzed to DP 2, as there is less steric hindrance from proximal branch points. According to the two models, *sbe1a *amylopectin contains a higher proportion of closely associated branch points than Wt. Furthermore, based on CL profiles (see Additional File 1 online), the calculated overall average branching density is similar in the two amylopectins. Thus, we suggest that the effect of the *sbe1a *mutation is to increase the local concentration of branch points but not to influence the overall amount of branch points in amylopectin.

In the debranched β-LDs from the amylose fraction (but not in intact amylose), a higher proportion of long chains of DP ≥ 100 was observed in *sbe1a *(Figure 1B and Additional File 1 online). The higher proportion of longer chains in β-LDs of amylose from *sbe1a *can be explained by branch points that tend to be closer to the non-reducing ends, so that longer internal chains result.

When debranching of β-LDs from amylose was performed with isoamylase without subsequent pullulanase digestion, there were fewer DP 2 than DP 3 chains (Figure 1B; see Additional File 4 online). For β-LD from amylopectin, all of the DP 3 and some of the DP 2 chains are known to be debranched by isoamylase [40]. However, our results of β-LD from amylose for both genotypes suggest that even some DP 3 chains are not debranched by isoamylase. Comparing *sbe1a *to Wt, more of DP 2 and DP 3 chains are not debranched by isoamylase in β-LD from *sbe1a *amylose (Figure 1B). As the structures escaping isoamylase debranching may have closely associated branch points and those structures can be debranched by pullulanase [40], a greater increase in both DP 2 and DP 3 by subsequent pullulanase treatment suggests that a higher proportion of these structures are resistant to isoamylase in amylose from *sbe1a*. Amylose branching pattern models are presented in Figure 5B to account for the difference in isoamylase action. In the model for *sbe1a*, A chains are preferentially attached by branch points close to each other whereas in Wt, A chains are not, leading to less hindered isoamylase debranching.

Our data suggest that amylose of *sbe1a *mutant starch has 1) longer internal chains and 2) more A chains attached by branch points close to each other. This evidence can be used to create an overall model for amylose branching patterns of *sbe1a *and Wt (Figure 5C). The models are drawn taking into account similar CL profiles (see Additional File 1 online) and assuming that ~50% of amylose molecules are branched, with ~5-6 branches per molecule [45]. According to the proposed model, for *sbe1a*, A chains are closer to each other, and the location of the chains tends to be more towards non-reducing end. For Wt, A chains are farther away from each other, and the location of the chains is more random and thus more distributed.

### Starch Digestion

Kinetic analysis shows that the *y_0 _*value for Wt starch is effectively zero (Table 1), in agreement with the RS value for Wt starch (1.6%), and the *y_0 _*and RS values for *sbe1a *starch are also in good agreement.

The kinetic model is based on the presence of two general types of starch substrate: a rapidly-digested substrate (*S*_*1*_), and a slowly-digested substrate (*S*_*2*_) [41,42]. The two genotypes differ both in the proportions of *S*_*1 *_and *S*_*2 *_and the reaction rate constants for these two components. The *S*_*1 *_components of Wt and *sbe1a *starch were 85.9% and 59.8% respectively. This suggests that the *sbe1a *mutation altered the starch structure and this resulted in less rapidly-digested component. Consistent with our results, Ao et al. [46] found that increased branch density led to a decreased proportion of RDS (analogous to our *S*_*1*_) and an increased proportion of SDS (analogous to our *S*_*2*_).

### Starch Granular Structure

Two microscopic techniques, scanning electron microscopy (SEM) and light microscopy (LM), were employed to observe granular structure before and after RS digestion by pancreatic α-amylase. Native starch granules from Wt and *sbe1a *appear similar in size, shape, degree of birefringence, and morphology, as described in a previous report for *wx *and *sbe1a wx *granules [47]. Polarized light microscopy (see Additional File 5 online) shows that almost all of the digested Wt granules had lost their birefringence, while for *sbe1a*, many digested granules had maintained some birefringence in the peripheral area of the granules, which indicates that the center of the digested *sbe1a *granules is either gone or no longer crystalline enough to show birefringence. The presence of a hollow interior in the digested *sbe1a *granules was confirmed by SEM (Figure 3B), indicating a relatively greater resistance to digestion for the exterior portion of the *sbe1a *granule.

Most of the recovered RS from Wt were represented by small granule fragments. However, the *sbe1a *RS showed variations in morphology, from small fragments to hollow granules. The difference in digestion of individual granules may be due to differences in heterogeneity in granule structure, as a higher proportion of relatively dark-stained granules were observed in *sbe1a *than in Wt native starch (Figure 3C). SEM revealed the presence of alternating layers in the Wt residual fragments (Figure 3B), which probably reflect the residual growth rings after digestion.

By observing the *sbe1a *RS by SEM (Figure 3B), one may roughly estimate that, for the recovered granules, approximately 40% of granule content has escaped digestion. However the RS value for *sbe1a *starch is approximately 13%. Therefore, some of the *sbe1a *granules were likely to have been digested completely. The heterogeneity found among *sbe1a *granules (Figure 3C) may account for different degree of digestion of individual granules. Thus, it can be reasoned that the micrographs of the *sbe1a *RS may disproportionately represent the more resistant granules.

A distinct feature of the recovered *sbe1a *RS is the presence of holes on the surface of the peripheral portion of the granules. These holes are possibly from the enlargement of the surface pores in native granules by α-amylase hydrolysis [48]. The presence of these holes on the shell is consistent with previous studies demonstrating that digestion of normal granules starts with surface pores and proceeds through deeper hydrolysis in channels [49-52], followed by fragmentation [48]. In the current study, the presence of remaining shells with holes in the *sbe1a *RS indicates continuing difficulty in digestion by α-amylase. Neither holes nor shells were observed in the Wt RS, indicating a more complete digestion.

As observed under microscopy, the RS from Wt consists mostly of portions of residual growth rings, while the RS of *sbe1a *is mostly residual peripheral regions. The kinetic analysis shows that the digestion of *sbe1a *starch reached a plateau by 16 h, suggesting the RS from *sbe1a *is not further digested. When the RS is observed by SEM, one can conclude that some of the peripheral regions in *sbe1a *starch granules cannot be further digested. Enrichment of amylose has been found by some to exist toward the granule peripheral region [53,54]. SEM showed that the peripheral regions were more resistant to α-amylase digestion in *sbe1a *granules. It is possible that these differences may be preferentially localized in the peripheral region of the granules, where starch synthesis may be more influenced by deficiency of SBEI [10]. The CL distribution of residual starch collected after α-amylase digestion showed some small differences between Wt and *sbe1a *(see Additional File 7 online). However, no direct evidence was obtained in the current study about whether the molecular structure in the peripheral regions was different in *sbe1a*.

### Starch Utilization during Kernel Germination

An endogenous α-amylase is considered to be responsible for attacking the starch granule and initiating starch hydrolysis in germinating cereal endosperm [55]. Starch hydrolysis continues by the action of limit dextrinase, α-amylase, β-amylase, and α-glucosidase to produce maltose and glucose for plant utilization [55]. The observed reduction in starch hydrolysis during the later stages of germination raises the possibility that continued hydrolysis of α-amylase-hydrolyzed glucans is hindered in the *sbe1a *mutant. The altered carbon metabolism could then cause a deficiency in general plant growth characteristics such as coleoptile length [23]. The structural analysis of *sbe1a *starch suggests that the decreased starch utilization of *sbe1a *seeds is due to an altered starch branching pattern.

### Consideration of SBEI Function in the Context of Pleiotropic Effects

Differences in SBE activity in *sbe *mutants could be simply due to the amount of a remaining SBE isoform or to biochemical or physical interactions that modulate the activities of an isoform; for the latter possibility SBEI may be regulated through complex interactions with other starch synthetic enzymes. Colleoni et al [21] showed that two migratory forms of SBEI are missing in maize endosperm of the maize *ae *mutant, indicating a possible interaction of SBEI and SBEIIb. Seo et al. [24] found that when SBEs were heterologously expressed in a yeast system, SBEIIa and/or SBEIIb appear to act before SBEI on synthesizing glucan structure. The studies of Yao et al. [25,26] suggest that in the absence of SBEIIb, a reciprocal inhibition exists between SBEI and SBEIIa, and that the presence of either SBEI or SBEIIa increases amylopectin branching as opposed to the presence of SBEI and SBEIIa together.

Direct evidence for protein-protein interactions between SBEs and different members of all the proteins involved in starch biosynthesis has also been reported by several groups, based on co-immunoprecipitation and affinity purification methods. Tetlow et al. [27] reported that SBEI from wheat amyloplasts was present in a high molecular weight complex with starch phosphorylase and SBEIIb. A separate study [56] using maize amyloplasts showed that eliminating SBEIIb caused significant increases in the abundance of SBEI, BEIIa, SSIII, and starch phosphorylase in the granule, without affecting SSI or SSIIa. Hennen-Bierwagen [30] reported that SBEI and SSI were shown to interact in one of three independent methods tested; SBEI did not interact with any of the other proteins in their study (SSIIa, SSIII, SBEIIa, SBEIIb), and unlike the other five proteins in their study, SBEI was the only protein to exist as a monomer in gel permeation chromatography.

In present study, the *sbe1a *mutant line is nearly isogenic with the Wt control. Most if not all mutant phenotypes are likely the result of many effects, direct and indirect, on the overall growth, development and physiology of the plant, so it is impossible to truly isolate a primary effect of the mutation when looking at a whole plant level phenotype, even the starch structure phenotype. Modifying SBE activity may induce modifications in the distribution of phosphate groups within amylopectin such as in potato [57,58]. This may alter accessibility of amylase (α or β) to its substrate and may induce differences in digestibility. Nonetheless, there is value in observing and characterizing the phenotype of these plants, both at the macro and molecular levels as we have presented. We have a sister paper [36] which does investigate the effect of various SBE mutations on leaf starch which further sheds light on the SBEI function in the context of pleiotropic consequences.

### Evolution and Function of Maize SBEI Isoform in Starch Biosynthesis

This work for the first time reports a specific and unique function for SBEI during the life cycle of maize. Molecular structure analysis suggests an important function of SBEI in modulating the branching pattern in normal starch by decreasing local clustering of amylopectin branch points. Thompson [59] emphasized the non-random nature of the distribution of branch points in starch. A specific type of non-random branching pattern may be required to optimize both storage and hydrolysis. It is reasonable to hypothesize that alteration in the specific non-random branching pattern could lead to an altered granule organization, rendering it more or less favorable to the plant for storage and/or for enzyme hydrolysis during utilization. Our data from *in vitro *starch digestibility and from plant germination analysis support this hypothesis.

Gene duplication and neo-functionalization are well known mechanisms by which specific genes can evolve to express different isoforms of enzymes with slightly specialized expression patterns or different enzymatic activities [60-62]. With the evidence from current and previous work, we can infer that an ancestral *Sbe *gene has duplicated at least twice during the evolution of maize, and these evolved to express three different SBE isoforms with highly specific functions in starch biosynthesis. A detailed phylogenetic analysis of the branching enzymes was published by Deschamps et al. [14]. This work demonstrated that genes belonging to both the SBEI and SBEII families can be identified in the green alga, which supports the theory that these two families of genes evolved approximately a billion years ago based on phylogenetic estimates of the divergence between the Chlorophyta and Magnolippyta lineages (estimates range from 729-1210 million years ago) [63,64]. This example of extreme evolutionary conservation is strong evidence for a specific and vital role for each enzyme isoform in starch biosynthesis. While most plant species studied retain genes representing each subfamily of SBE, Arabidopsis does not, suggesting that somewhere in the lineage leading to Arabidopsis, the gene was lost with minimal consequences to the species [65].

The evidence presented in this work strongly supports the hypothesis that SBEI is required to synthesize endosperm starch granules that allow normal hydrolysis and utilization during germination. Considering plant survival in the wild, optimal seedling vigor would be a strong evolutionary force to select for genotypes of plants with starch granules optimized for molecular structure that would lead to efficient storage and utilization. The reduced seedling vigor of *sbe1a *mutant seeds observed in this work provides powerful evidence for a specialized and important role of SBEI in plant development, consistent with the evolutionary conservation of SBEI in all higher plants.

## Conclusions

This work for the first time reports that a lack of SBEI activity resulted in an observable effect, which was seen on both starch molecular structure and starch function. Structural and functional analysis of endosperm starch deficient in SBEI activity strongly supports the hypothesis that SBEI is required to synthesize starch granules for normal kernel development, allowing efficient hydrolysis and utilization.

Evidence from this work reveals a unique and essential function of SBEI in normal plant development, consistent with the evolutionary conservation of SBEI in all higher plants.

The new knowledge generated in this work will contribute to our understanding of the function and evolution of the maize SBEs, and of their roles in the biosynthesis, hydrolysis and utilization of starch granules. Moreover, the novel *sbe1a *starch might have application as a food ingredient with nutritional benefit.

## Methods

### Starch Material

Maize plants of Wt and *sbe1a *mutant were grown during summer, 2007 at Penn State Horticultural Research Farm (Rock Springs, PA). In order to compare starch material within a highly similar genetic background, homozygous *Sbe1a/Sbe1a *(i.e. Wt) and *sbe1a/sbe1a *mutant siblings were identified from a single segregating population derived from seeds of selfed *Sbe1a/sbe1a *plants to obtain ears for endosperm analysis. Genotyping of Wt and *sbe1a *mutant plants followed Blauth et al. [37]. The detected homozygous Wt and *sbe1a *mutant plants were self-pollinated to produce ears for endosperm analysis, and are segregated from a BC_4_F_3 _population backcrossed by Blauth et al. [11,37]. Starch extraction from three different ears, considered as three biological replications, for each genotype, was according to Yao et al. [66]. Starch fractionation followed Klucinec and Thompson [67].

### β-Amylolysis of Amylopectin and Debranching of β-Dextrins

β-Dextrins were prepared by the method of Xia and Thompson [40] with slight modifications in sample size. Amylopectin samples (48 mg) were dispersed in 480 μL of 90% dimethyl sulfoxide (DMSO) by heating in a boiling water bath for 10 min. To the dispersion, warm sodium acetate buffer (3.52 mL, 50°C 0.02*M*, pH 6.0) was added. The mixture was heated in a boiling water bath for 10 min and cooled to 50°C. A 200-μL aliquot of a β-amylase (from barley, Cat.No. E-BARBL; Megazyme International Ireland, Ltd.) solution (250 U/mL, 0.02*M *sodium acetate, pH 6.0) was added, and the samples were incubated at 50°C with constant agitation (200 strokes/min). At approximately 10 min, 30 min, 1 h, 2 h, 6 h, and 24 h, a 0.5-mL aliquot of sample was removed and heated in a boiling water bath for 10 min to stop the reaction. The procedures for precipitating β-dextrins and debranching β-dextrins by successive action of isoamylase (from *Pseudomonas *sp., Cat.No. E-ISAMY; Megazyme) and pullulanase (from *Klebsiella planticola*, Cat.No. E-PULKP; Megazyme) were the same as used previously for β-LDs) [39,40].

### Preparation of Isoamylase-Debranched and Isoamylase plus Pullulanase-Debranched β-Limit Dextrins from Amylose Fractions

The preparation and debranching of β-LDs followed the procedures in Klucinec and Thompson [39] with slight modifications in sample size. After the β-LDs were debranched with isoamylase for 24 h, a 30-μL aliquot of the digested solution was added to 270 μL of DMSO and reserved for analysis by high-performance size-exclusion chromatography (HPSEC). Then the β-LDs were further debranched with pullulanase for 24 h, afterwards another 30-μL aliquot of the digested solution was added to 270 μL of DMSO for HPSEC analysis [40].

### Resistant Starch Determination

The official method for *in vitro *RS determination (AOAC 2002.02, AACC 32-40) was employed, which was scaled-down and modified for direct analysis of the digestion supernatant for total carbohydrate [41]. The modification allowed analysis of digestion time-course for small starch samples (~20 mg). For RS determination, after the 16 h digestion step at 37°C with porcine pancreatic α-amylase and amyloglucosidase (enzymes from RS Assay Kit, Cat.No. K-RSTAR, Megazyme), the sample tube was removed from the water bath and to an aliquot of each sample was added 1 volume of 95% (v/v) ethanol with 0.5% (w/v) EDTA. After centrifugation (1,500 × *g*, 10 min), the supernatant was analyzed in duplicate for total carbohydrate using the phenol sulfuric acid method [68]. The percent non-digested starch (% NDS) was calculated from this data and was the basis for the calculation of the RS value. Starch isolated from Wt and *sbe1a *mutant endospermes from three separate plants (triplicate biological replications) were subjected to triplicate pancreatic α-amylase digestion, for determining the RS values.

### Digestion Time-Course Analysis

For determination of digestion time-course, the starch samples were digested as described above. An aliquot was removed at approximately 30 sec, 3 min, 6 min, 15 min, 30 min, 1 h, 2 h, 3 h, 4 h, 5 h, 7 h, 10 h, 13 h, and twice at 16 h, and added to 1 volume of ethanol/EDTA solution to ensure immediate deactivation of the enzymes. After centrifugation the supernatants were analyzed for total carbohydrate as described above.

Digestion time-course was analyzed following the method developed by Rees [42] to obtain kinetic data. A "Double, 5 parameter" regression model in SigmaPlot (Systat Software, Inc.) was selected to fit the data using the double exponential decay equation:

where y is % NDS, x is the time, *y*_*0 *_is the *y*-value that the model asymptotically approaches, *S*_*1 *_and *S*_*2 *_are the concentrations of the two different substrate components, and *k*_*1 *_and *k*_*2 *_are the reaction rate constants for the decay of the two different components. The units for *y*_*0*_, *S*_*1*_, and *S*_*2 *_were % of initial starch, and the units for the rate constants were min^-1^. After running the regression program, the software gives three possible completion status messages depending on how well the model fits the data:

(1) Converged, tolerance satisfied.

(2) Converged, tolerance satisfied. Parameter may not be valid. Array numerically singular on final iteration.

(3) Didn't converge, exceeded maximum number of iterations.

The data were kept for further regression analysis if message 1 or 2 resulted, and were discarded if message 3 resulted.

Digestion time-course analysis was performed for three biological replications per genotype. For each biological replication, two technical replications were performed. If both sets of data "converged" using the model (message 1 or 2), no further analyses were performed. If message 3 appeared, a new technical replication was done until the data "converged." The data from the two "converged" technical replications for each biological replication were combined, and the software program was run on the combined data. For all samples, the regression model fit for the combined data completed with convergence (Message 1), and generated valid parameters for analysis. Using the combined data, values for five parameters in the equation were determined for each biological replication. A mean and standard deviation of the five parameters for each genotype was then calculated, and comparisons among genotypes were made by one-way ANOVA analysis.

### Light Microscopy

Bright field and polarized light microscopy were performed using a light microscope (BX51; Olympus) with an attached digital camera (Spot II RT; Diagnostic Instruments). 5 mg of native starch sample was mixed with 0.5 mL of deionized water in a micro-centrifuge tube. For the resistant starch samples, the supernatant was removed after centrifugation of digestion solution and 20 μL of deionized water was added to the pellets to disperse the sample. To examine the sample under the microscope, 20 μL of the dispersed sample was added to a glass slide, and a cover slip was fixed over the sample with fingernail polish. Examination of iodine-stained starch followed the method in Evans and Thompson [54]. 20 μL of iodine solution (0.08% I_2_, 0.12% KI) was placed onto 20 μL of the dispersed sample to give a final I_2 _concentration of 0.04%. In order to compare birefringence between granules, the camera's automatic exposure function was turned off, and the exposure was set the same for all samples. The same sample field was examined under bright field and polarized light.

Heterogeneity of iodine staining was evaluated quantitatively by a volunteer panel. Differentially iodine-stained starch granules were classified into two categories, dark or light stained granules, and were sorted visually by five individual evaluators who were not otherwise involved in the research. The evaluators were trained to understand the difference between dark and light stained granules, by observing granules in a portion of a micrograph for *sbe1a *granules. Four micrographs for each genotype (Wt or *sbe1a*) were used for sorting. The evaluators were then given those eight micrographs, unlabeled and in randomized order, and asked to sort the granules into two categories. The proportion of dark granules for each micrograph was calculated based on the sorting results from all five evaluators, and a mean proportion was obtained for each micrograph. For each genotype, a mean was calculated from the means of the four micrographs. Comparison between two genotypes was made by one-way ANOVA.

### Scanning Electron Microscopy

A thin layer of starch sample was applied to double-sided sticky carbon tape on a specimen stub, and sputter-coated with 10 nm Au/Pd (BAL-TEC SCD 050; US-TechnoTrade). Samples were then examined using a scanning electron microscope (JSM-5400; JEOL Ltd.) at an accelerating voltage of 20 keV and at different magnification levels (1,500 ×, 3,500 ×, and 10,000 ×). For image collection, lower magnification was first employed to examine the whole view of samples, and higher magnification was then used to focus on sample areas that were representative overall.

### Kernel Germination Assay

A kernel germination assay was performed according to the method in Dinges et al. [23] with slight modifications. Mature, dried maize kernels were surface-sterilized by immersion in 15 mL of 1% sodium hypochlorite for 5 min and then washed three times with deionized water. 15 kernels from each of three ears for each genotype were placed in Petri dishes containing three layers of moist Whatman paper and incubated at 30°C in the dark. The length of each coleoptile was measured by a ruler on successive days throughout the 11-day incubation period. To measure the amount of endosperm starch remaining, the roots, coleoptiles, embryo, and pericarps were removed from 2 kernels at days 1, 6, 8, and 11. The remaining endosperm was ground with a mortar and pestle on ice. The powered tissue was washed into a tube with deionized water and homogenized with a Tissumizer (Model SDT 1810; Tekmar) at 20,000 rpm for 1 min. The ground tissue was washed with deionized water, centrifuged at 1500 × *g *for 10 min, and suspended in 3 mL of deionized water. For calculating the dry weight of samples, 1 mL of this suspension was dried at 70°C overnight and weighed. The remaining 2 mL of the suspension was boiled for 30 min, and the total glucan polysaccharide in the solubilized solution was quantified in triplicates, using a commercial assay kit that measures glucose released after digestion with α-amylase and amyloglucosidase (Cat.No. K-TSTA; Megazyme). The quantified starch content was normalized against the dry weight for comparison between genotypes.

## Authors' contributions

HX conceived of the study, performed the experiments, and drafted the manuscript. MYN participated in maize genotype breeding and discussion of experimental design and major results. DBT & MJG advised the conception of the study, experimental design, result discussion, and revised the manuscript. All authors read and approved the final manuscript.

## Supplementary Material

Additional file 1 **Chromatograms of isoamylase-debranched amylopectin and amylose fractions from Wt (----) and *sbe1a *mutant (- - -) starch**. Click here for file

Additional file 2 **Difference plots between *sbe1a *mutant and Wt starch for the proportions of chains from debranched β-dextrins during time course of β-amylolysis of amylopectin**. Individual plots for *sbe1a *mutant and Wt are presented in Figure 1A. Click here for file

Additional file 3 **Chain length distribution of isoamylase-debranched and isoamylase-plus-pullulanase-debranched β-limit dextrins from the amylopectin fraction from Wt and *sbe1a *mutant starch**. Click here for file

Additional file 4 **Chain length distribution of isoamylase-debranched and isoamylase-plus-pullulanase-debranched β-limit dextrins from the amylose fraction from Wt and *sbe1a *mutant starch**. Click here for file

Additional file 5 **Bright field (left) and polarized light (right) micrographs of residual starch after 16 h α-amylase digestion from Wt and *sbe1a *mutant**. Arrows point to residual granules with dark center. Click here for file

Additional file 6 **Transmission electron micrographs of residual starch after 16 h α-amylase digestion from Wt (left) and *sbe1a *mutant (right)**. Scale bars represent 5 μm at the top of the graphs. Click here for file

Additional file 7 Chromatograms of isoamylase-debranched resistant starch from Wt (----) and *sbe1a *mutant (- - -) starch Click here for file
