# Supplementary material for: Deficiency of maize starch-branching enzyme i results in altered starch fine structure, decreased digestibility and reduced coleoptile growth during germination
Source: BMC Plant Biol. 2011 May 21;11:95. doi: 10.1186/1471-2229-11-95 (PMC3245629; doi:10.1186/1471-2229-11-95)
Supplement: Additional file 1 — Chromatograms of isoamylase-debranched amylopectin and amylose fractions from Wt (----) and sbe1a mutant (- - -) starch. [file 1471-2229-11-95-S1.PDF]

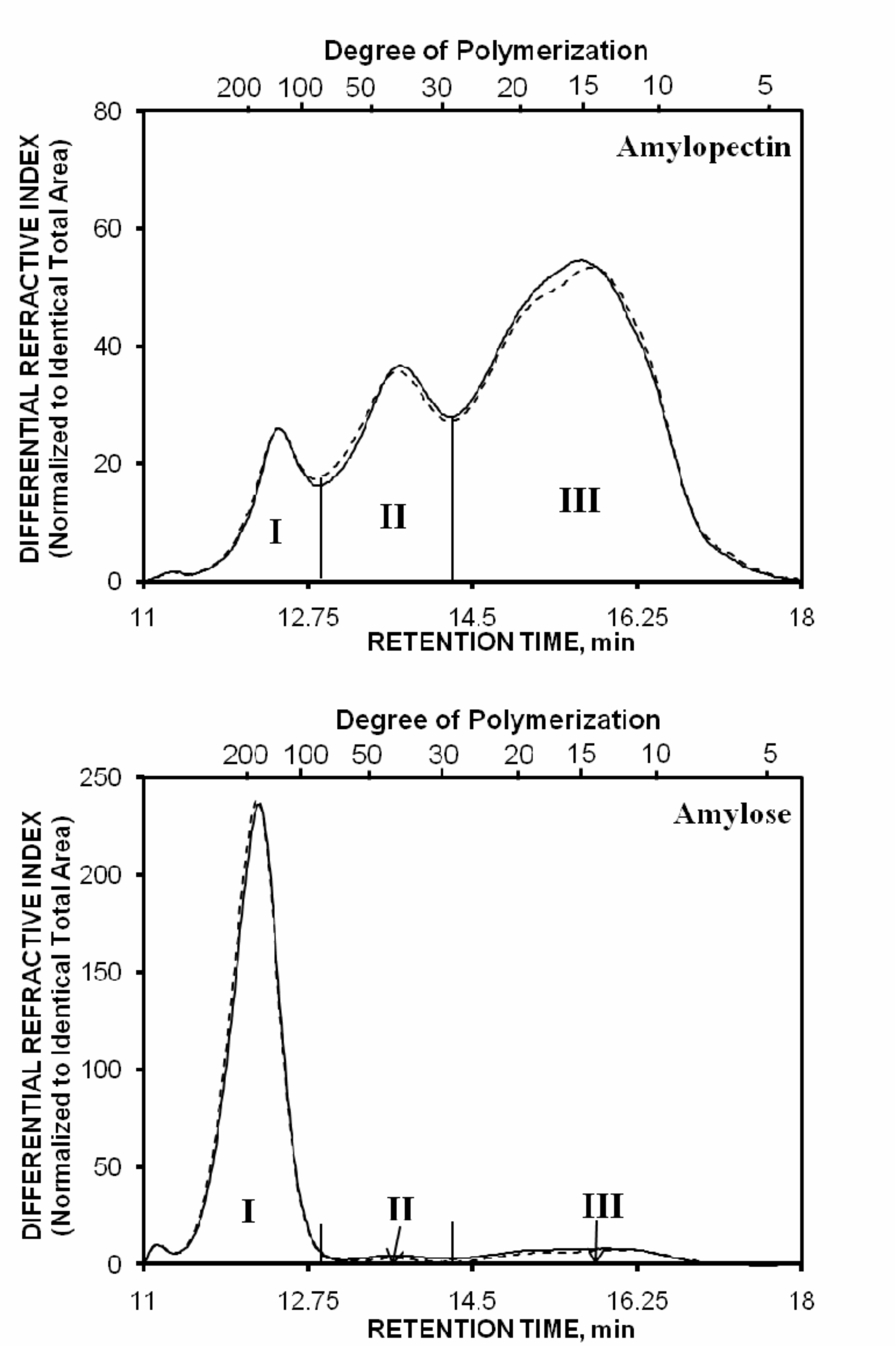

Additional File 1. Chromatograms of isoamylase-debranched amylopectin and amylose fractions from Wt (——) and *sbe1a* mutant (– – –) starch[1].

[1]*Representative chromatograms for starch from one biological replication.*
