# Supplementary material for: Deficiency of maize starch-branching enzyme i results in altered starch fine structure, decreased digestibility and reduced coleoptile growth during germination
Source: BMC Plant Biol. 2011 May 21;11:95. doi: 10.1186/1471-2229-11-95 (PMC3245629; doi:10.1186/1471-2229-11-95)
Supplement: Additional file 2 — Difference plots between sbe1a mutant and Wt starch for the proportions of chains from debranched β-dextrins during time course of β-amylolysis of amylopectin. Individual plots for sbe1a mutant and Wt are presented in Figure 1A. [file 1471-2229-11-95-S2.PDF]

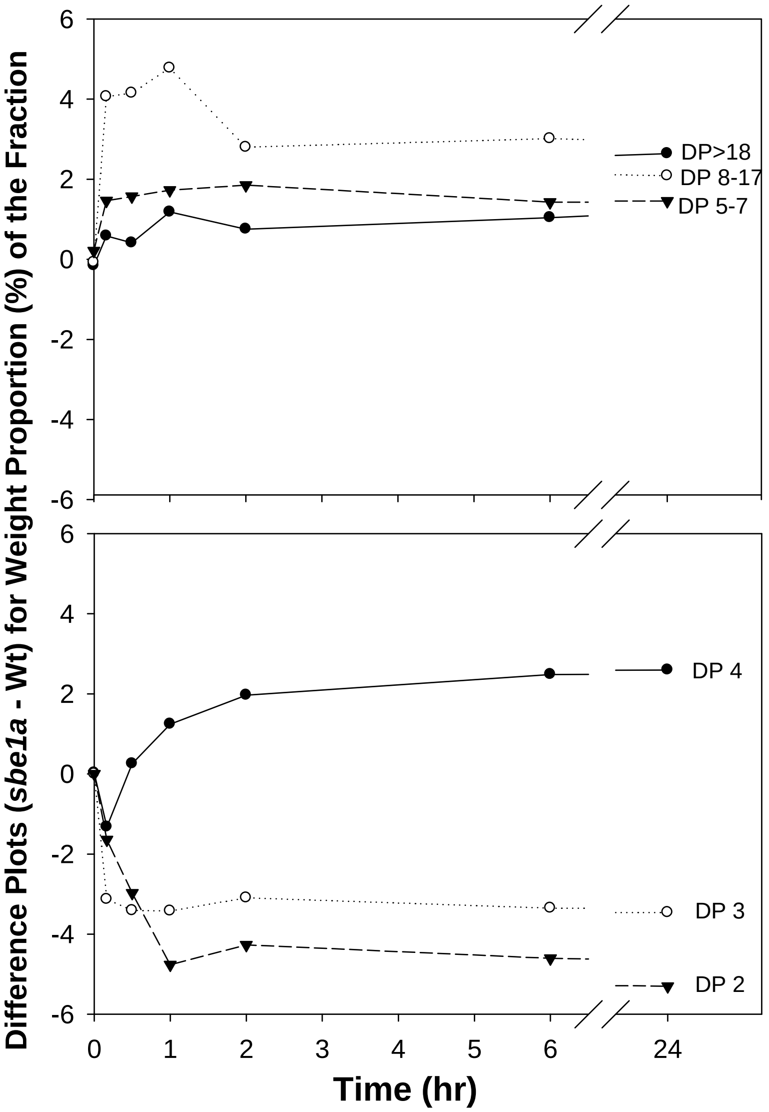

Additional File 2. Difference plots between *sbe1a* mutant and Wt starch for the proportions of chains from debranched β-dextrins during time course of β-amylolysis of amylopectin. Individual plots for *sbe1a* mutant and Wt are presented in Fig. 1A.
