# Supplementary material for: Deficiency of maize starch-branching enzyme i results in altered starch fine structure, decreased digestibility and reduced coleoptile growth during germination
Source: BMC Plant Biol. 2011 May 21;11:95. doi: 10.1186/1471-2229-11-95 (PMC3245629; doi:10.1186/1471-2229-11-95)
Supplement: Additional file 3 — Chain length distribution of isoamylase-debranched and isoamylase-plus-pullulanase-debranched β-limit dextrins from the amylopectin fraction from Wt and sbe1a mutant starch. [file 1471-2229-11-95-S3.PDF]

Additional File 3. Chain length distribution of isoamylase-debranched and isoamylase-plus-pullulanase-debranched β-limit dextrins from the amylopectin fraction from Wt and *sbe1a* mutant starch[1].

**β-Limit Dextrin from Amylopectin**[3]	**Chromatographic Region**[2]			
	**DP 5-7**	**DP 4**	**DP 3**	**DP 2**
**Wt**				
Isoamylase	8.9 ± 0.3[b]	2.5 ± 0.3[a]	6.9 ± 0.3[a]	3.1 ± 0.3[c]
Isoamylase, then pullulanase	9.5 ± 0.2[c]	2.7 ± 0.3[a]	7.2 ± 0.4[a]	6.4 ± 0.4[d]
Increase by Pullulanase	0.6 ± 0.5[a]	-	-	3.3 ± 0.1[c]
sbe1a				
Isoamylase	9.5 ± 0.3[c]	6.1 ± 0.6[b]	6.8 ± 0.6[a]	2.1 ± 0.3[b]
Isoamylase, then pullulanase	9.6 ± 0.4[c]	6.1 ± 0.5[b]	6.8 ± 0.7[a]	3.2 ± 0.0[c]
Increase by Pullulanase	-	-	-	1.1 ± 0.3[a]

[1]*Values are percentage by weight. Values are mean ± standard deviation based on two independent analyses for one biological replication. Significant differences in the same column,* as *determined by one-way ANOVA with Fisher's LSD multiple comparison procedure, are indicated by different superscripts.*

[2]*Proportions of DP 5–7, DP 4, DP 3 and DP 2 were calculated as the areas for* $4.5 \leq DP \leq 7.5$, $3.5 \leq DP \leq 4.5$, $2.5 \leq DP \leq 3.5$, *and* $DP \leq 2.5$, *respectively, as in [40].*

[3]*The β-limit dextrins from amylopectin were either debranched by isoamylase, or by isoamylase plus pullulanase, indicated by "Isoamylase" and "Isoamylase, then pullulanase", respectively.*
