# Supplementary material for: Deficiency of maize starch-branching enzyme i results in altered starch fine structure, decreased digestibility and reduced coleoptile growth during germination
Source: BMC Plant Biol. 2011 May 21;11:95. doi: 10.1186/1471-2229-11-95 (PMC3245629; doi:10.1186/1471-2229-11-95)
Supplement: Additional file 4 — Chain length distribution of isoamylase-debranched and isoamylase-plus-pullulanase-debranched β-limit dextrins from the amylose fraction from Wt and sbe1a mutant starch. [file 1471-2229-11-95-S4.PDF]

Additional File 4. Chain length distribution of isoamylase-debranched and isoamylase-plus-pullulanase-debranched β-limit dextrins from the amylose fraction from Wt and *sbe1a* mutant starch[1].

β-Limit Dextrins from Amylose[3]	Chromatographic Region[2]						
	B_L Chains		B_S Chains		Bs or A chains	A chains	
	DP ≥ 100	DP 18-99	DP 8-17	DP 5-7	DP 4	DP 3	DP 2
**Wt**							
Isoamylase	40.3 ± 2.7[a]	22.4 ± 0.9[b]	23.5 ± 2.3[b]	5.2 ± 0.5[d]	1.1 ± 0.1[b]	3.4 ± 0.1[d]	4.1 ± 0.7[c]
Isoamylase, then pullulanase	38.3 ± 5.8[a]	23.5 ± 1.0[b]	23.3 ± 2.7[b]	4.8 ± 0.9[c,d]	1.0 ± 0.8[b]	3.9 ± 0.1[e]	5.4 ± 0.5[d]
Increase by pullulanase	-	-	-	-	-	0.4 ± 0.2[a]	1.3 ± 0.2[a]
sbe1a							
Isoamylase	57.3 ± 4.1[b]	17.2 ± 1.5[a]	17.3 ± 2.4[a]	3.5 ± 0.3[b]	0.6 ± 0.0[a]	2.2 ± 0.0[c]	2.0 ± 0.4[b]
Isoamylase, then pullulanase	55.6 ± 3.4[b]	16.2 ± 1.0[a]	15.7 ± 2.5[a]	4.0 ± 0.1[c]	1.0 ± 0.2[b]	3.2 ± 0.3[d]	4.3 ± 0.3[c]
Increase by pullulanase	-	-	-	0.6 ± 0.4[a]	0.4 ± 0.2[a]	0.9 ± 0.3[b]	2.3 ± 0.1[b]

[1]*Values are percentage by weight. Values are mean ± standard deviation based on two independent analyses for one biological replication. See Figure 1B. Significant differences in the same column,* as *determined by one-way ANOVA with Fisher's LSD multiple comparison procedure, are indicated by different superscripts.*

[2]*Proportions of DP ≥ 100, DP 18-99, DP 8-17, DP 5–7, DP 4, DP 3 and DP 2 were calculated as the areas for DP ≥ 99.5, 17.5 ≤ DP ≤ 99.5, 7.5 ≤ DP ≤ 17.5, 4.5 ≤ DP ≤ 7.5, 3.5 ≤ DP ≤ 4.5, 2.5 ≤ DP ≤ 3.5, and DP ≤ 2.5, respectively, as in [40].*

[3]*The β-limit dextrins from amylose were either debranched by isoamylase, or by isoamylase plus pullulanase, indicated by "Isoamylase" and "Isoamylase, then pullulanase", respectively.*
