# Supplementary material for: Deficiency of maize starch-branching enzyme i results in altered starch fine structure, decreased digestibility and reduced coleoptile growth during germination
Source: BMC Plant Biol. 2011 May 21;11:95. doi: 10.1186/1471-2229-11-95 (PMC3245629; doi:10.1186/1471-2229-11-95)
Supplement: Additional file 5 — Bright field (left) and polarized light (right) micrographs of residual starch after 16 h α-amylase digestion from Wt and sbe1a mutant. Arrows point to residual granules with dark center. [file 1471-2229-11-95-S5.PDF]

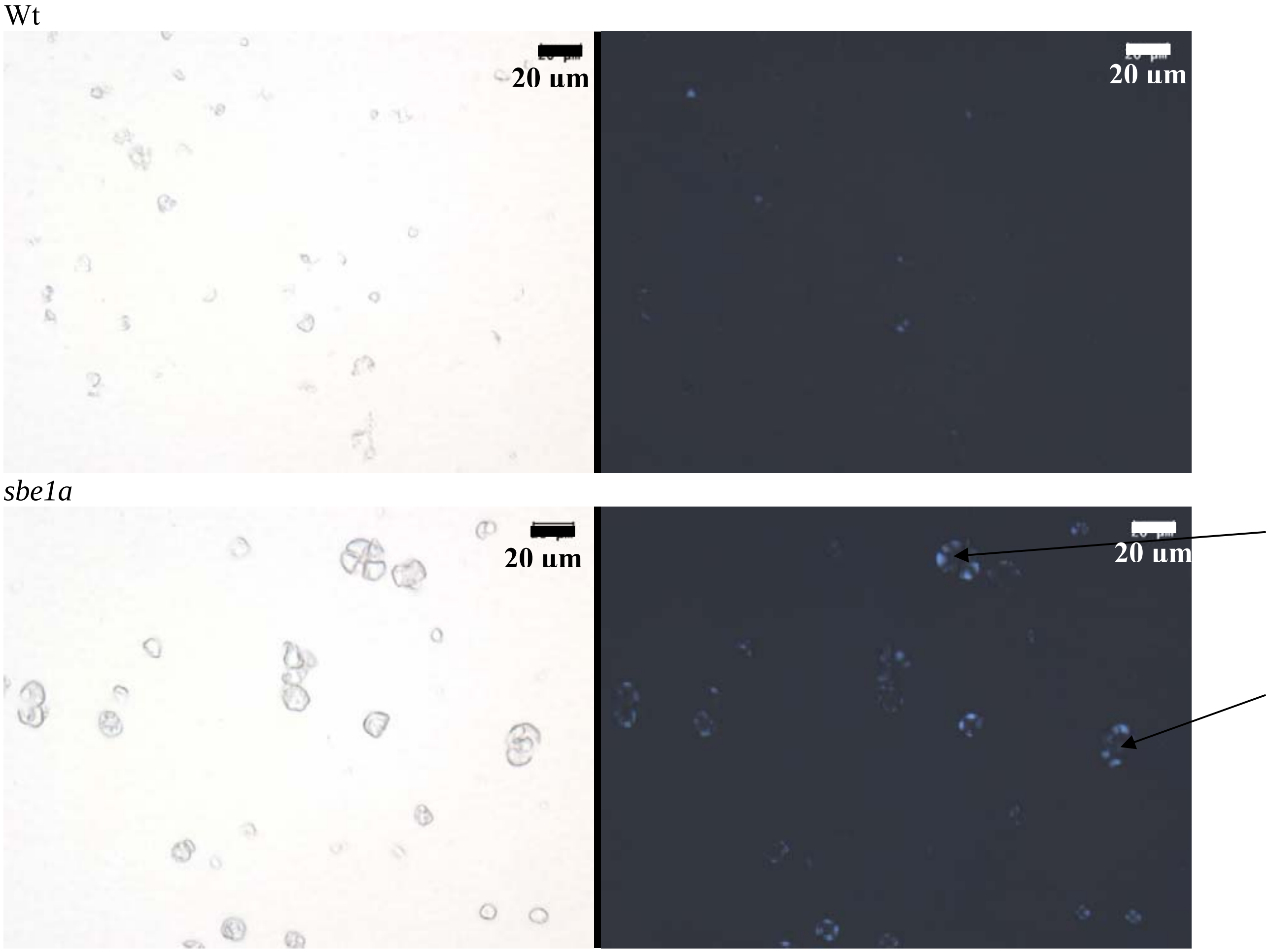

Additional File 5. Bright field (left) and polarized light (right) micrographs of residual starch after 16 h α-amylase digestion from Wt and *sbe1a* mutant. Arrows point to residual granules with dark center.
