# Supplementary material for: Deficiency of maize starch-branching enzyme i results in altered starch fine structure, decreased digestibility and reduced coleoptile growth during germination
Source: BMC Plant Biol. 2011 May 21;11:95. doi: 10.1186/1471-2229-11-95 (PMC3245629; doi:10.1186/1471-2229-11-95)
Supplement: Additional file 6 — Transmission electron micrographs of residual starch after 16 h α-amylase digestion from Wt (left) and sbe1a mutant (right). Scale bars represent 5 μm at the top of the graphs. [file 1471-2229-11-95-S6.PDF]

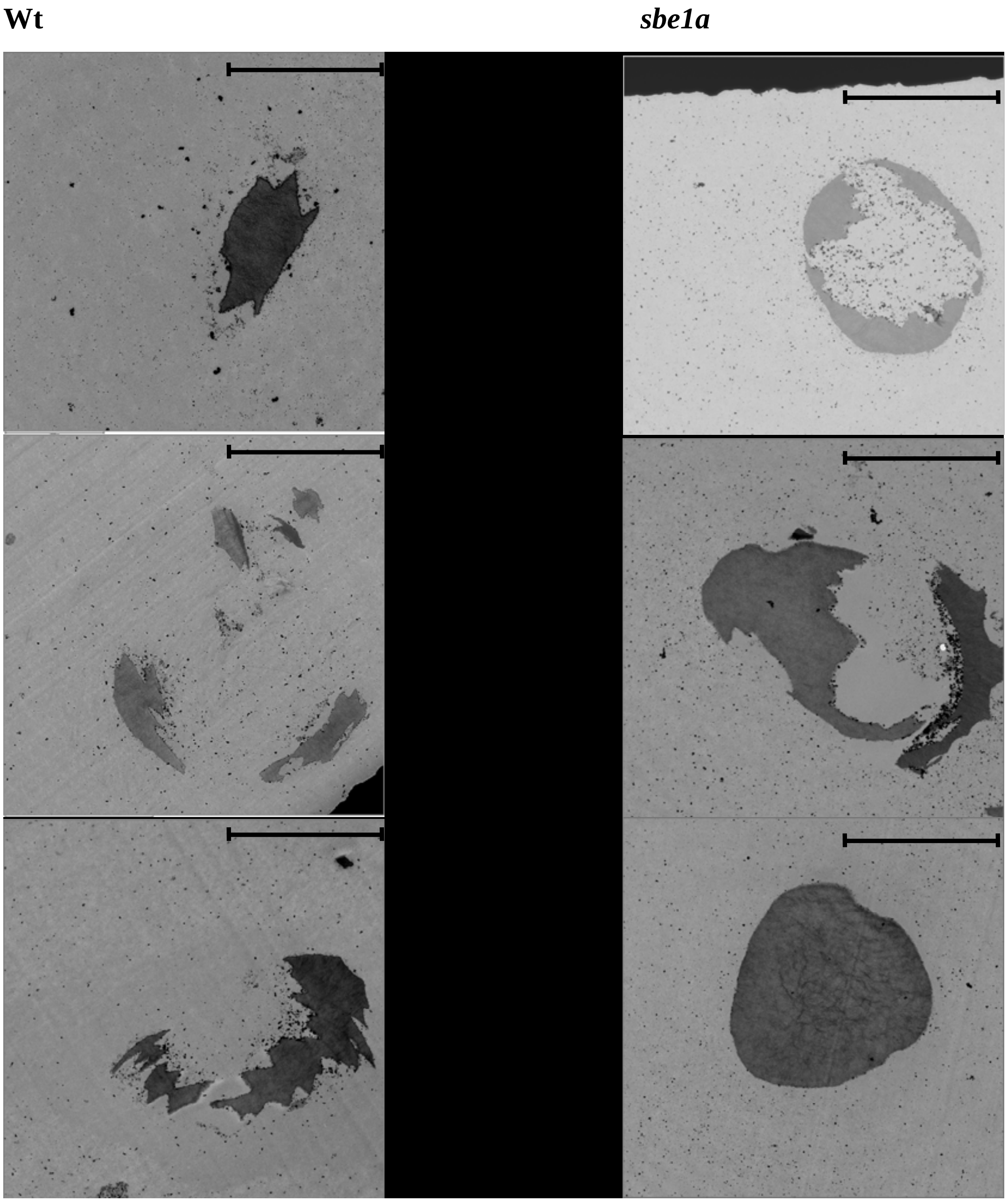

Additional File 6. Transmission electron micrographs of residual starch after 16 h α-amylase digestion from Wt (left) and *sbe1a* mutant (right). Scale bars represent 5 μm at the top of the graphs.
